# New York Letter

**Published:** 1895-11

**Authors:** 


### NEW YORK LETTER.

NEW YORK, September 1, 1895.

In September your correspondent visited the new site, at Waverley, Massachusetts, of the well-known McLean Hospital for the treatment of the insane, which has been located in Somerville for three-quarters of a century.

The new buildings were almost ready for occupancy, and the removal of the patients was to be made the last of September, or the first of October, the old grounds having been purchased by the Boston & Lowell Railroad Company, which was to have possession by that time.

Three years ago, the trustees purchased the present site, at Waverley, consisting of 176 acres of land beautifully situated on rising ground, giving a wide view to the south and east, overlooking Boston and the harbor and neighboring cities.

With the change in location, the institution has also changed its name from "asylum," so long an objectionable form, to "hospital," which is a decided improvement.

The new grounds form a beautiful park with walks and drives and wide stretches of lawn. The main group of buildings, eight in number, occupies the center of the grounds and consists of the administration building, flanked on either side, at a distance of 125 to 250 feet apart, by the buildings for patients, three on one side for men and four on the other for women. These are connected by asphalted walks, beneath which are covered ways which connect the buildings by the basement. Each building, architecturally, has an individuality, though they are all of a colonial style, and each covers an area of from 5,000 to 9,000 square feet.

Back of this group are situated the buildings for the boilers and dynamos for furnishing the heat and electric lighting, kitchens, laundries, etc., and also two gymnasiums, one for the women and one for the men.

At a distance of some seven hundred feet is another building for men, the Upham Memorial, built by Mr. George P. Upham in memory of his son. This building has its own heating apparatus in the basement, is a beautiful, large building in colonial style, with *porte--cochère*, a broad covered porch, and an entrance into a wide, spacious hall. It contains a reception and dining rooms and nine suites of apartments, each consisting of a parlor, bedroom, and bath. It also has a billiard room and rooms for Turkish bath in the basement, with rooms for nurses and attendants at the top.

The administration building contains the quarters for the hospital staff and their offices, meeting rooms for the trustees, a library and large entertainment hall for the patients. Back of this is a laboratory for chemistry, pathological and microscopical work.

A little apart is the house of the superintendent, Dr. Cowles, and at one end of the grounds a building has been erected to be used as a convalescent home for patients, from the Massachusetts General Hospital.

Of course, the very latest improvements in the care of the insane are to be found here, and the improvements that have taken place in the last three-fourths of a century, the duration of the institution, may well be imagined. That the separation of the insane into smaller buildings instead of one large one, has been found to be more conducive to their well-being, goes without saying, and is well worth the increased cost of administration.

The writer desires to thank Dr. George T. Tuttle and Dr. D. H. Fuller, the first and second assistant physicians, for their courtesy towards him in showing him over the buildings and explaining the workings of the institution.

The long looked for changes in the hospitals in charge of the Commissioners of Charities, have at last been made, and as was bound to be, when such sweeping changes are made, much injustice has been done. The offices of visiting physicians and surgeons of all the hospitals under their charge, except Bellevue, were abolished. This is to take place November 1st. This included City and Maternity Hospitals, Hospital for Nervous Diseases, the Workhouse, Almshouse and Hospital for Incurables (these are all on Blackwell's Island), Randall's Island Hospital, and Gouverneur and Fordham Hospitals. These positions were given over to the medical schools—Physicians and Surgeons, Bellevue, and University, and also the so-called Fourth Division of Bellevue, each having an equal number of appointments to make.

Several members of the old staffs were reappointed, but the majority were left out, and men who had worked with these institution for years were dismissed without a recognition of their services.

Already, protests are heard, and resolutions were passed at the annual meeting of the State Medical Association, condemning the manner in which the medical schools made their appointments in the case of Harlem Hospital, and, at the last meeting of the County Medical Association, resolutions were passed, strongly protesting against this method of treating medical men. Undoubtedly, we shall hear of still others. In the long run, however, whatever injustice, for the moment, has been done individual medical men, the change will prove a good one, unless the next turn of the wheel should bring back the old regime. We believe that the power of appointment has been placed where it should be, and that a body of cultivated and educated physicians, even though they are not entirely free from petty enmities and jealousies, are far better capable of judging the merits of a candidate for a vacancy than the party boss, a ward boss, or ward heeler. The old methods of appointment at these institutions had long been one of the many scandals connected with the manner in which they have been under political domination. If, in the future, the hospital supplies and food and accommodations for patients shall improve in the same proportion as the method of choosing the visiting staffs, these places will be far more eagerly sought than they have been in the past.

A physician, member of the County Medical Society, has a lawsuit on his hands for $40,000, brought against him by an irate optician, who alleges this damage to his business, because said physician objected to his advertising in the Medical Directory published by the County Medical Society and endeavored to have it removed in the future; being unsuccessful in the endeavor, he called the attention of the Society to it in a paper. He applied to the Comitia Minora for support in the lawsuit against him; but the committee declined to be responsible for his utterances, and he appealed to the Society at large, and a committee of five was appointed to investigate and report to the Society. And at the last meeting, held October 28th, the Society voted by an overwhelming majority to assist the physician in his suit, thus reversing the policy of the Comitia Minora.

The New York State Medical Association held its annual meeting in this City, October 15th, 16th, and 17th. Among the papers read was one by Dr. W. H. Park on "Recent Studies on Diphtheria and Pseudo-Diphtheria," showing the mortality rate in the past year, since the use of antitoxine, as compared with previous years. In 5,777 cases treated with antitoxine in the hospitals, the mortality was 18.7 per cent., and in previous years, in these same hospitals, there was an average rate of 43.6 per cent. In private practice, in 663 cases, the mortality was 6.6 per cent.

In cases of laryngeal diphtheria, few in proportion demanded operative relief, and in the case of those demanding relief, the mortality was 38 per cent. with antitoxine; as compared with previous years, a mortality of 70 per cent.

In New York City, the mortality rate of all cases reported for four years preceding the use of antitoxine was 34 per cent., for the past nine months it was 17 per cent.

Papers on malignant growths were numerous. Dr. W. B. Coley presented six patients with detailed histories and the histories of two more, eight in all, successfully treated for inoperable growths, by means of mixed toxins of erysipelas and bacillus prodigissus. The president, Dr. Austin Flint, made the annual address, taking for his subject, "The Coming Role of the Medical Profession in the Scientific Treatment of Crime and Criminals."

The daily press is taking more notice than formerly of medical affairs, reporting papers read at the Academy of Medicine. And one ambitious band of newsgatherers has been sending attending surgeons of hospitals a typewritten circular, requesting them to send word or report of interesting cases and their methods employed, promising that a reporter will be sent and that their names shall be used, and requesting them not to be so "fossilized" in this twentieth century as to hide their light under a bushel.

